# PLIN2 is a Key Regulator of the Unfolded Protein Response and Endoplasmic Reticulum Stress Resolution in Pancreatic β Cells

**DOI:** 10.1038/srep40855

**Published:** 2017-01-19

**Authors:** Elaine Chen, Tsung Huang Tsai, Lan Li, Pradip Saha, Lawrence Chan, Benny Hung-Junn Chang

**Affiliations:** 1 Department of Molecular & Cellular Biology, Baylor College of Medicine, Houston, TX, USA; 2 Department of Medicine, Division of Diabetes, Endocrinology & Metabolism, Diabetes Research Center, Baylor College of Medicine, 1 Baylor Plaza, Houston, TX 77030, USA.

## Abstract

Progressive pancreatic β cell failure underlies the transition of impaired glucose tolerance to overt diabetes; endoplasmic reticulum (ER) stress expedites β cell failure in this situation. ER stress can be elicited by lipotoxicity and an increased demand for insulin in diabetes. We previously reported that the lipid droplet protein perilipin 2 (PLIN2) modulates lipid homeostasis in the liver. Here, we show that PLIN2 modulates the unfolded protein response (UPR) and ER stress in pancreatic β cells. PLIN2 expression goes up when β cells are exposed to a lipid load or to chemical ER stress inducers. Downregulation of PLIN2 ameliorates the effects of fatty acid- and chemical-induced ER stress, whereas PLIN2 overexpression exacerbates them. Diabetic Akita mice, which carry a heterozygous C96Y *Ins2* mutation, exhibit elevated PLIN2 expression and ER stress in their β cells. Genetic ablation of *Plin2* in Akita mice leads to mitigation of ER stress, forestalling β cell apoptosis, partially restoring β cell mass, and ameliorating diabetes. Mechanistic experiments showed that PLIN2 downregulation is associated with enhanced autophagic flux and accelerated ER stress resolution. In sum, we have identified a crucial role for PLIN2 in modulating autophagy, ER stress resolution, and β cell apoptosis and survival.

The pancreatic β cell devotes ~50% of its protein synthetic capacity towards the production of proinsulin/insulin^1^. In type 2 diabetes (T2D), the increased insulin production presents a challenge for the endoplasmic reticulum (ER)^2^, where the folding of newly synthesized proinsulin/insulin occurs. ER stress arises when misfolded proteins accumulate inside the ER lumen, triggering the unfolded protein response (UPR). The UPR preempts or alleviates ER stress by increasing chaperone protein expression to increase ER folding capacity, decreasing mRNA translation, and stimulating ER-associated degradation. When UPR fails to relieve ER stress, pancreatic β cell apoptosis ensues^2^,^3^. Elevated ER stress markers are known to occur in the pancreatic islets of patients with diabetes^4^,^5^,^6^.

Elevated plasma non-esterified fatty acids (NEFA) commonly occur in people with T2D^7^. NEFA are stored in cells as triglyceride (TG) and other specialized lipids enveloped by a monolayer of phospholipids in an organelle called lipid droplet (LD). In addition to resident lipids, numerous proteins reside on the surface of LDs to control cellular lipid homeostasis. The perilipins (PLINs) constitute the major LD proteins (LDPs) that encompass five members, PLIN1 to PLIN5. Of these, PLIN2 is a constitutively expressed LDP that occurs in essentially all cells^8^, including hepatocytes and pancreatic β cells^9^. In the liver, PLIN2 level goes up with lipid load^10^,^11^,^12^, and ablation of PLIN2 lowers hepatic TG content^13^,^14^. Exposure of β cells to fatty acids stimulates PLIN2 expression, whereas downregulation of PLIN2 prevents fatty acid-induced TG accumulation^15^. Prolonged exposure to fatty acids also induces ER stress in β cells that often culminates in their apoptosis^4^,^16^,^17^,^18^. However, the role of PLIN2 in modulating ER stress in pancreatic β cells remains unexplored.

In this study, we first examined the role of PLIN2 in the UPR of pancreatic β cells when they are exposed to NEFA. We then dissected PLIN2 function in the glucose homeostatic response of pancreatic β cells in Akita mice, which house a cysteine-to-tyrosine (C96Y) *Ins2* gene mutation. An analogous *INS* mutation causes monogenic diabetes in humans^19^,^20^,^21^. This mutation precludes proper disulfide bond formation, causing proinsulin to misfold, and preventing it from maturation and secretion^22^,^23^. Akita mice display severe ER stress, failure of glucose-stimulated insulin secretion in their β cells, and diabetes. We found that PLIN2 regulates ER stress in β cells of Akita mice, and that induced downregulation of PLIN2 mitigates the ER stress response and protects β cells from apoptosis. Moreover, we showed that the protective effect of loss of PLIN2 is mediated at least in part by upregulated autophagy in the β cell.

## Methods

### Animals

We purchased Akita mice in C57BL/6 background from The Jackson Laboratory^22^. We generated *Plin2*^−/−^ mice as described previously^13^ and obtained Akita;*Plin2*^−/−^ mice by crossing Akita mice with *Plin2*^−/−^ mice. All experiments were performed in accordance with relevant guidelines and regulations approved by the Institutional Animal Care and Use Committee of Baylor College of Medicine.

### Metabolic measurements

We incubated isolated pancreases in acid-ethanol (0.2 N HCl in 70% ethanol) overnight at 4 °C, and homogenized them the following day. After centrifugation, we neutralized the solution to pH 7.2 with 0.4 M Tris pH 8.0 buffer. Insulin levels were then measured with an insulin ELISA kit (Mercodia). For glucose tolerance test, we injected 1.5 g of glucose/kg body weight intraperitoneally.

### Cell culture and reagents

We obtained Akita β cell line and wild-type control cell line from Dr. Akio Koizumi^24^. Akita, control, and MIN6 β cells were cultured in DMEM (Cellgro) containing 4.5 g/L glucose, 15% FBS (Life Technologies), penicillin (100 U/ml), streptomycin (100 μg/ml), and β-mercaptoethanol (150 μM) (Sigma-Aldrich). For fatty acid treatment, we incubated cells in 1% BSA (Sigma-Aldrich) alone, BSA conjugated-oleic acid, or -palmitic acid. To induce ER stress, we treated pancreatic islets or other cells with 0.1% DMSO alone, tunicamycin, or thapsigargin (Sigma-Aldrich).

### Immunoblotting

We isolated total proteins in RIPA lysis buffer supplemented with protease and phosphatase inhibitor cocktail tablets (Roche). Proteins were separated on 4–15% gradient gels (Bio-Rad) and transferred to PVDF membranes. We blocked membranes with 5% BSA-TBST and incubated them with primary antibodies overnight at 4 °C. The membranes were washed and incubated with secondary antibodies conjugated with horseradish peroxidase (Bio-Rad) on the following day, and visualized using chemiluminescence system (Millipore).

### Quantitative real-time PCR

We isolated total RNA using the Trizol reagent (Life Technologies) and used iScript cDNA Synthesis Kit (Bio-Rad) for reverse-transcription. cDNA was used as a template for quantitative PCR with SYBR Green-Rox mix (Quanta BioSciences) on an Mx3000 P thermal cycler (Agilent). We calculated relative expression levels of transcripts vs. housekeeping genes using the ΔΔCT method.

### Statistical analysis

Data were presented as mean ± SEM. Results were examined for statistical significance by ANOVA followed by post hoc analysis. A *p*-value less than 0.05 was considered significant different.

## Results

### PLIN2 regulates lipid homeostasis in pancreatic β cells

**Figure 1:**
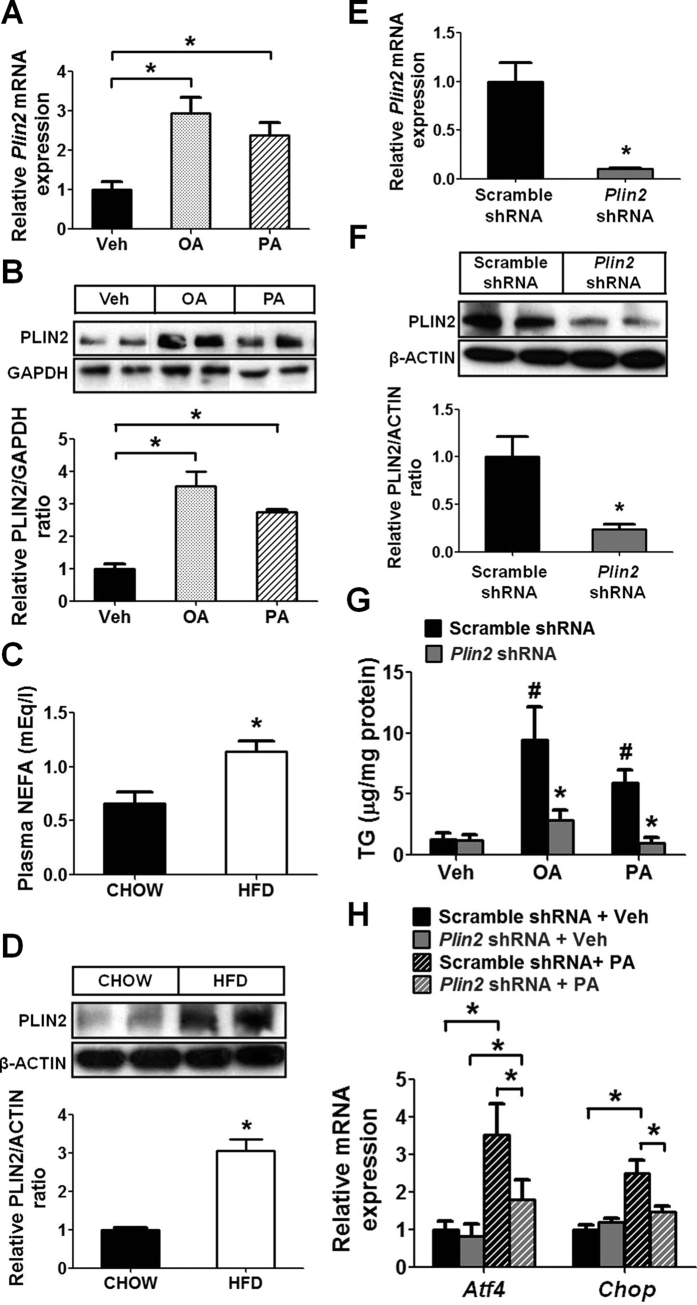
PLIN2 regulates lipid homeostasis and NEFA-induced ER stress in pancreatic β cells. (**A**,**B**) MIN6 cells were treated with vehicle, 0.4 mM oleic acid (OA), or 0.4 mM palmitic acid (PA) for 16 hours (n = 4–6). (**A**) Quantitative RT-PCR. (**B**) Immunoblotting and quantification. GAPDH was used as a loading control. (**C**,**D**) C57BL/6 mice were fed with normal chow or 40% high fat diet (HFD) for 12 weeks (n = 6). (**C**) Plasma non-esterified fatty acids (NEFA). (**D**) Immunoblotting and quantification for PLIN2 protein in pancreatic islets. β-ACTIN was used as a loading control. (**E**,**F**) MIN6 cells were transduced with scramble-shRNA or *Plin2*-shRNA lentivirus. *Plin2*-knockdown was examined by (**E**) quantitative RT-PCR (n = 4) and (**F**) immunoblotting. β-ACTIN was used as a loading control. (**G**,**H**) MIN6 cells were treated the same as panels (A,B) (n = 5). (**G**) Quantification of triglycerides (TG). TG levels were normalized to total protein. *p < 0.05 in comparison with respective scramble; ^#^*p* < 0.05 in comparison with vehicle. (**H**) Quantitative RT-PCR. All immunoblotting quantification was normalized to vehicle/control. *p < 0.05.

Exposure of MIN6, a mouse insulinoma cell line, to oleic acid (OA) or palmitic acid (PA) increased PLIN2 expression in these cells (Fig. 1A and B). To determine if PLIN2 expression is modulated by NEFA *in vivo*, we fed C57BL/6 mice a high fat diet (HFD) for 12 weeks. Compared to mice on regular chow, mice on HFD had elevated plasma NEFA (Fig. 1C) as well as increased PLIN2 protein in their pancreatic islets (Fig. 1D).

We next examined the role of PLIN2 in NEFA-induced ER stress. We generated stable *Plin2*-knockdown MIN6 cells using shRNA-lentivirus and confirmed the knockdown efficiency by quantitative RT-PCR and immunoblotting (Fig. 1E and F). The *Plin2*-knockdown cells displayed markedly lower TG on exposure to OA and PA, which raised the cellular TG ~8 and ~6 folds, respectively, in control cells (Fig. 1G). It was previously reported that saturated NEFA were more potent than unsaturated NEFA in inducing ER stress^17^,^18^; we therefore examined whether PA induces ER stress in MIN6 cells. We found that PA increased *Atf4* and *Chop*, the downstream targets of UPR transducer PERK (Fig. 1H). Notably, the PA-stimulated *Atf4* and *Chop* upregulation was blunted in *Plin2*-knockdown cells (Fig. 1H), indicating that reduced PLIN2 expression ameliorates NEFA-induced ER stress in pancreatic β cells.

### Chemical-induced ER stress upregulates PLIN2 expression in pancreatic β cells

**Figure 2:**
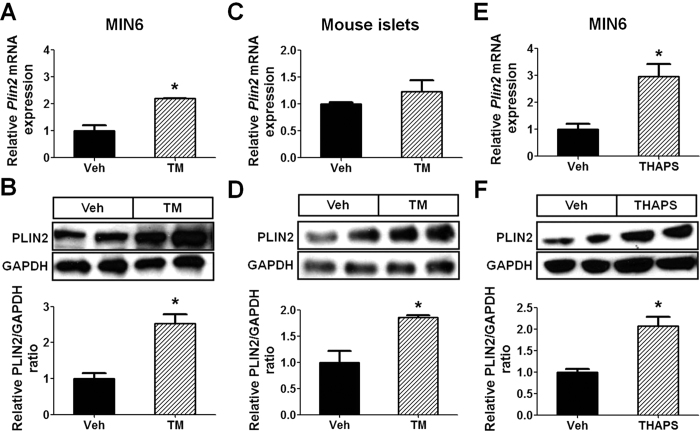
ER stress upregulates PLIN2 expression in MIN6 cells and mouse pancreatic islets. (**A**,**B**) MIN6 cells and (**C**,**D**) isolated C57BL/6 mouse pancreatic islets were treated with vehicle or 5 μg/ml tunicamycin (TM) for 6 hours (n = 4–6). (**A**,**C**) Quantitative RT-PCR and (**B**,**D**) immunoblotting and quantification for PLIN2 expression. GAPDH was used as a loading control. (**E,F**) MIN6 cells treated with vehicle or 5 μM thapsigargin (THAPS) for 6 hours (n = 4–6). (**E**) Quantitative RT-PCR and (**F**) immunoblotting and quantification for PLIN2 expression. GAPDH was used as a loading control. All immunoblotting quantification was normalized to vehicle. *p < 0.05.

We treated MIN6 cells with ER stress inducer tunicamycin (TM) and found that the treatment increased PLIN2 expression at both the mRNA and protein levels (Fig. 2A and B). The same treatment also stimulated PLIN2 protein expression (and a trend towards increased *Plin2* mRNA expression) in isolated mouse islets (Fig. 2C and D). In addition to TM, thapsigargin (THAPS), an ER stress inducer that works via different mechanisms^25^, also stimulated PLIN2 expression in MIN6 cells (Fig. 2E and F). The results suggest that increased ER stress *per se* is associated with upregulated PLIN2 expression.

### Downregulation of PLIN2 protects against chemical-induced ER stress in β cells

**Figure 3:**
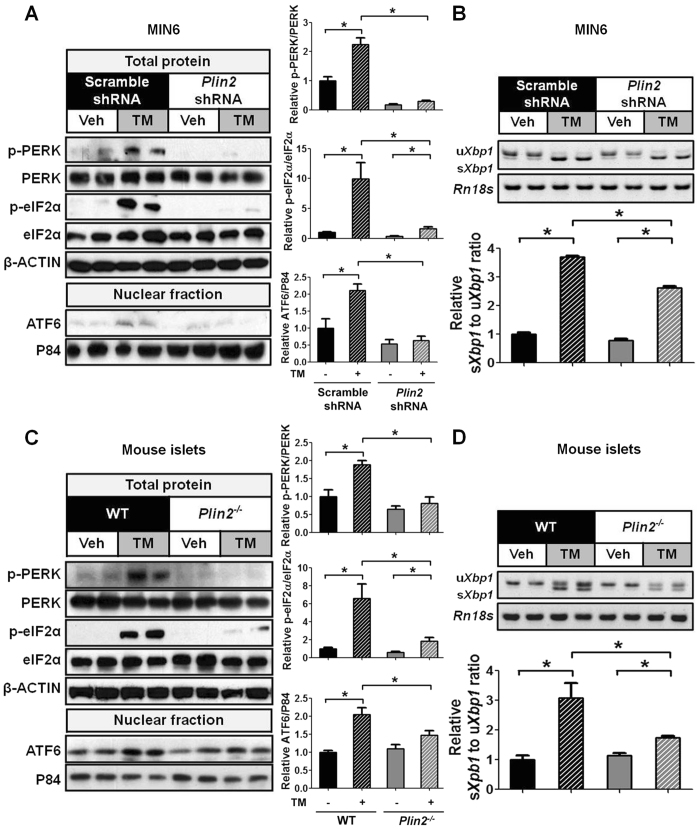
Downregulation of PLIN2 protects against ER stress in MIN6 cells and mouse pancreatic islets. (**A**,**B**) MIN6 cells and (**C**,**D**) pancreatic islets of wild-type (WT) and *Plin2*^−/−^ mice were treated with vehicle or 5 μg/ml tunicamycin (TM) for 6 hours. (**A**,**C**) Immunoblotting and quantification for p-PERK, PERK, p-eIF2α, eIF2α, and nuclear ATF6 protein. β-ACTIN and P84 were used as loading controls for total and nuclear proteins respectively. (**B**,**D**) Semi-quantitative RT-PCR and quantification results for spliced *Xbp1* (s*Xbp1*) and unspliced *Xbp1* (u*Xbp1*) mRNA. *Rn18s* was used as a loading control. Quantification was normalized to scramble-shRNA vehicle or WT vehicle. *p < 0.05.

We hypothesized that reduced PLIN2 level attenuates ER stress and its downstream effects. We first treated control and *Plin2*-knockdown MIN6 cells with TM and assayed for molecules that inform all three branches of the UPR. We found that TM stimulated the levels of p-PERK, p-eIF2α (PERK pathway), and nuclear ATF6 protein (ATF6 pathway) (Fig. 3A) and upregulated the ratio of s*Xbp1* to u*Xbp1* mRNA (IRE1α pathway) (Fig. 3B) in control MIN6 cells. In contrast, TM-induced activation of the PERK pathway was significantly blunted in *Plin2*-knockdown MIN6 cells (Fig. 3A). The ratio of s*Xbp1* to u*Xbp1* mRNA and the nuclear ATF6 protein levels were also attenuated in *Plin2*-knockdown MIN6 cells (Fig. 3A and B).

We next isolated pancreatic islets from *Plin2*^−/−^ and wild-type mice and treated them with TM. Corroborating the results using MIN6 cells, we found that the levels of p-PERK, p-eIF2α, nuclear ATF6 proteins (Fig. 3C), and of s*Xbp1* mRNA were significantly attenuated in islets isolated from *Plin2*^−/−^ compared with wild-type mice (Fig. 3D). Taken together, the results indicate that partial or complete deficiency of PLIN2 attenuates TM-induced UPR signaling pathways in β cells.

### PLIN2 accumulation enhances ER stress of pancreatic β cells

**Figure 4:**
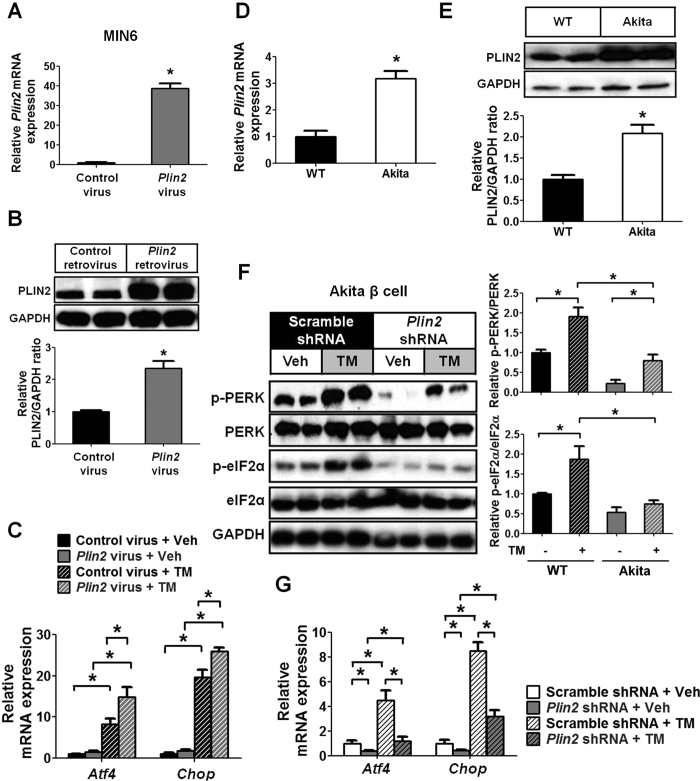
PLIN2 accumulation enhances the ER stress of β cells. (**A**,**B**) MIN6 cells were transduced with control or *Plin2*-retrovirus. PLIN2 overexpression was examined by (**A**) quantitative RT-PCR (n = 4) and (**B**) immunoblotting and quantification. GAPDH was used as a loading control. (**C**) Quantitative RT-PCR. Control and *Plin2*-overexpression MIN6 cells treated with vehicle or 5 μg/ml tunicamycin (TM) for 6 hours (n = 4–6). (**D**,**E**) Total RNA and protein were collected from control wild-type (WT) β cells and Akita β cells (n = 5–6). PLIN2 expression was examined by (**D**) quantitative RT-PCR and (**E**) immunoblotting and quantification. GAPDH was used as a loading control. (**F**,**G**) Control and *Plin2*-knockdown Akita β cells were treated with vehicle or 5 μg/ml TM for 6 hours (n = 4–6). (**F**) Immunoblotting and quantification. GAPDH was used as loading control. (**G**) Quantitative RT-PCR. All immunoblotting quantification was normalized to vehicle/control. *p < 0.05.

To determine if PLIN2 overproduction *per se* affects the TM-stimulated UPR in pancreatic β cells, we generated MIN6 cells that stably overexpress PLIN2 by retrovirus transduction. The upregulated PLIN2 expression was confirmed by quantitative RT-PCR and immunoblotting (Fig. 4A and B). Interestingly, gene transduction-induced PLIN2 overexpression further augmented the TM-stimulated *Atf4* and *Chop* responses in these cells (Fig. 4C), indicating that PLIN2 overexpression *per se* leads to an exaggerated UPR in pancreatic β cells.

### Effect of alteration of PLIN2 expression on the UPR

We next sought to determine if the increased ER stress in the pancreatic β cells of Akita mice is also associated with changes in PLIN2 expression. We found that, indeed, PLIN2 expression was upregulated in Akita β cells compared to controls (Fig. 4D and E). *Plin2*-knockdown Akita cells displayed attenuated p-PERK and p-eIF2α expression compared with control Akita cells under basal conditions and after TM (Fig. 4F). There was also a significant attenuation of the mRNA expression of *Atf4* and *Chop* in *Plin2*-knockdown Akita β cells with and without TM treatment (Fig. 4G). Thus, induced PLIN2 deficiency significantly downregulates the exaggerated UPR in Akita β cells.

### PLIN2 ablation alleviates hyperglycemia in Akita mice

**Figure 5:**
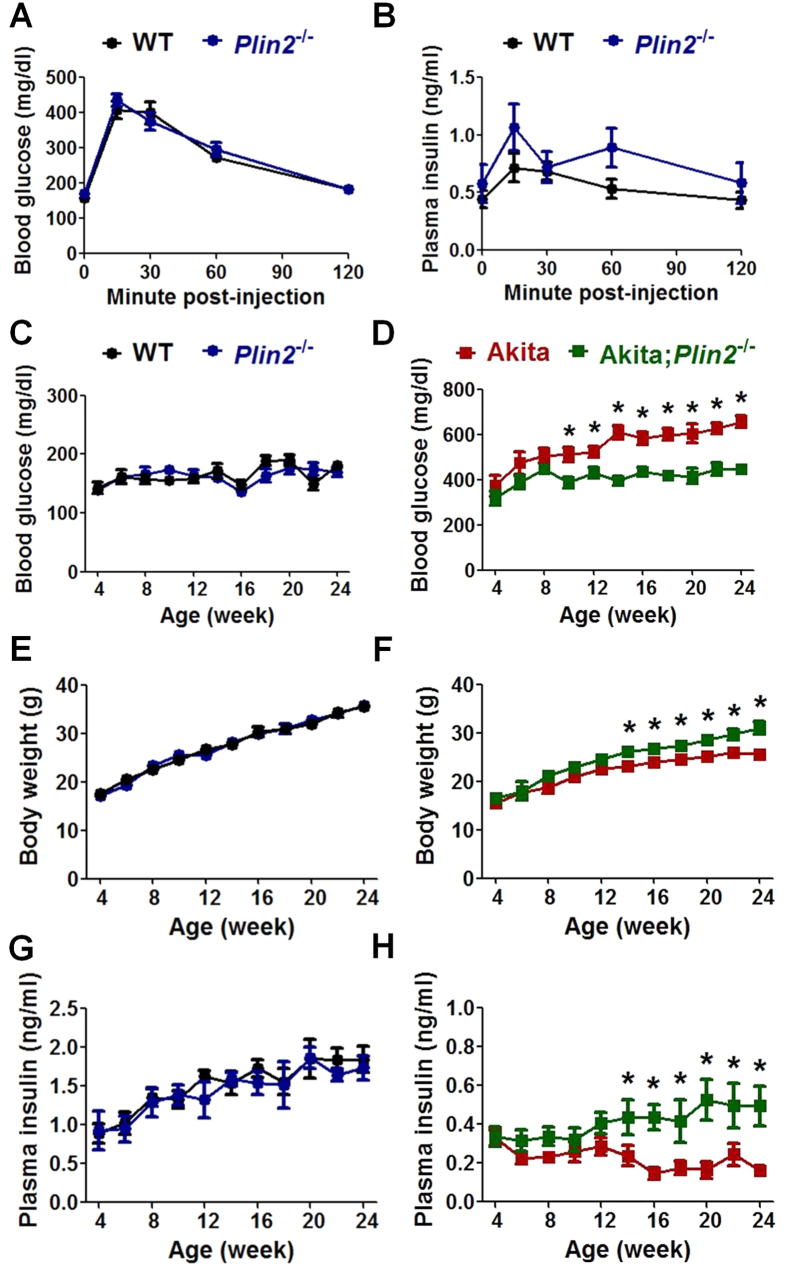
PLIN2 ablation alleviates hyperglycaemia in Akita mice. (**A**) Blood glucose and (**B**) plasma insulin levels at designated time points during intraperitoneal glucose tolerance test of 14-week-old male mice (n = 7–10). Mice were fasted for 4 hours in the morning prior to injection with 1.5 g glucose/kg body weight. (**C**,**D**) Random morning blood glucose levels, (**E**,**F**) body weight, and (**G**,**H**) random morning plasma insulin levels at indicated age (n = 6–10). *p < 0.05.

We bred Akita mice into *Plin2*^−/−^ mice and compared the glucose homeostasis of wild-type, *Plin2*^−/−^, Akita, and Akita;*Plin2*^−/−^ mice. Glucose tolerance test (GTT) in 14-week-old *Plin2*^−/−^ and wild-type mice revealed no significant difference in plasma glucose or insulin between the two genotypes (Fig. 5A and B). We next measured random morning blood glucose levels and found no difference between *Plin2*^−/−^ and wild-type mice (Fig. 5C). In agreement with previous studies^22^,^26^, we observed that Akita mice developed hyperglycemia at ~8 weeks of age. In contrast, the hyperglycemia was significantly attenuated in Akita;*Plin2*^−/−^ mice (Fig. 5D). There was no difference in the body weights of wild-type and *Plin2*^−/−^ mice (Fig. 5E). Akita mice showed considerable lag in weight gain compared with wild-type mice. Loss of PLIN2 expression partially rescued the failure to gain weight in Akita mice (Fig. 5E and F). Akita mice also displayed significantly lower random morning plasma insulin than wild-type. Notably, the plasma insulin of Akita;*Plin2*^−/−^ mice was consistently higher than that of Akita mice (Fig. 5H). There was no difference in plasma insulin concentrations between wild-type and *Plin2*^−/−^ mice during the same time period (Fig. 5G). Moreover, ablation of *Plin2* improved the GTT response in Akita mice (Fig. S1A). Collectively, these data indicate that the loss of PLIN2 partially rescues the insulin deficiency and hyperglycemia of Akita mice.

### Deletion of PLIN2 partially rescues β cell mass in Akita mice

**Figure 6:**
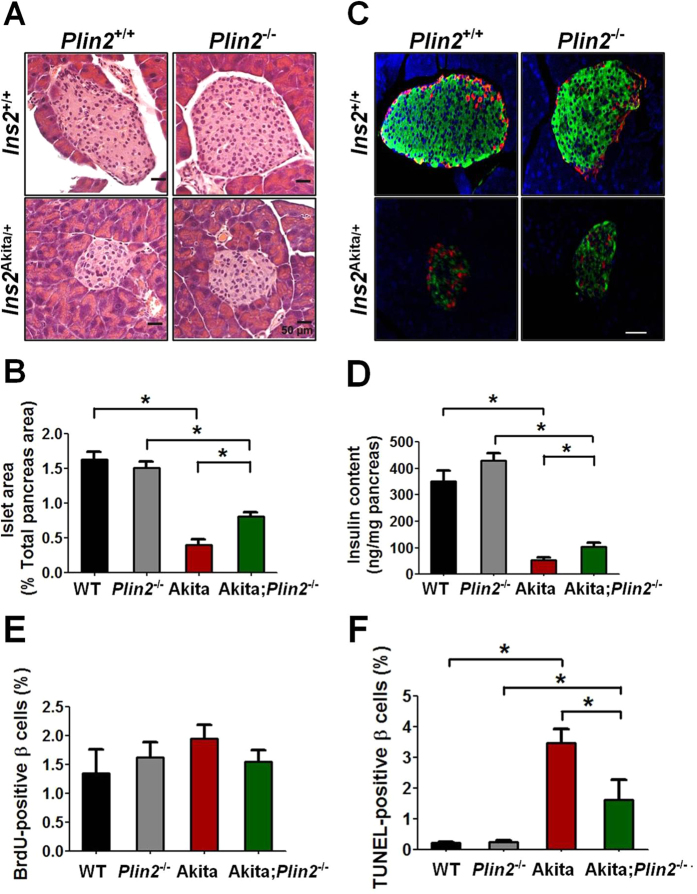
Ablation of PLIN2 partially rescues β cell mass in Akita mice. (**A**) Hematoxylin and eosin staining of pancreas sections from 14-week-old mice. (**B**) Quantification of the islet area relative to the pancreas area. Total islet and pancreas area were counted from 6 sections per mouse (n = 5). (**C**) Insulin (green) and glucagon (red) immunofluorescence staining of pancreas sections from 14-week-old mice. Nuclei were stained with DAPI (blue). (**D**) Total pancreas insulin content of 14-week-old mice (n = 7). (**E**) Quantification of BrdU-positive β cells and (**F**) TUNEL-positive β cells. Percent BrdU-positive and TUNEL-positive β cells were calculated from 6 sections per mouse (n = 5). *p < 0.05. Scale bar = 50 μm.

Histological examination of tissue sections showed that presence or absence of PLIN2 did not affect the size of pancreatic islets in mice with wild-type background (Fig. 6A). In confirmation of previous studies^26^, Akita mice had much smaller islets than wild-type. In comparison, the islets of Akita;*Plin2*^−/−^ mice were significantly larger than those in Akita mice (Fig. 6A). Quantification of the ratio of islet to total pancreas area from pancreas sections revealed that *Plin2*^−/−^ and wild-type mice displayed similar total islet area. The area in Akita mice was ~25% of wild-type; loss of PLIN2 in Akita;*Plin2*^−/−^ mice led to a doubling in islet area (Fig. 6B). Immunofluorescence staining showed morphologically indistinguishable islets from wild-type and *Plin2*^−/−^ mice, whereas Akita mice had much smaller islets that stained poorly with insulin antibody. Loss of PLIN2 in Akita mice partially rescued these defects. Quantification of the ratio of insulin-positive islet area to total pancreas area showed that ablation of *Plin2* in Akita;*Plin2*^−/−^ mice partially rescued the β cell mass (Fig. S1B). Quantification of the insulin content in pancreas extracts of mice of different genotypes revealed that the presence or absence of PLIN2 had no effect in wild-type mice (Fig. 6D). In contrast, pancreatic insulin content was markedly reduced in Akita mice, and loss of PLIN2 partially reversed the insulin deficiency (Fig. 6D).

We quantified the BrdU-positive cells in the islets of mice and found that the four genotypes exhibit similar proportions of BrdU-positive cells (Fig. 6E). We then examined the proportion of apoptotic cells and found a very low level of TUNEL-positive cells in wild-type and *Plin2*^−/−^ islets (Fig. 6F). Whilst Akita β cells displayed an increased number of TUNEL-positive cells than wild-type β cells, loss of PLIN2 in Akita;*Plin2*^−/−^ mice reduced the proportion of apoptotic cells in Akita mice (Fig. 6F). Therefore, the relative preservation of β cell mass and insulin content in Akita;*Plin2*^−/−^ mice appears to be mainly a result of downregulation of apoptosis.

### PLIN2 modulates autophagic flux in pancreatic β cells

**Figure 7:**
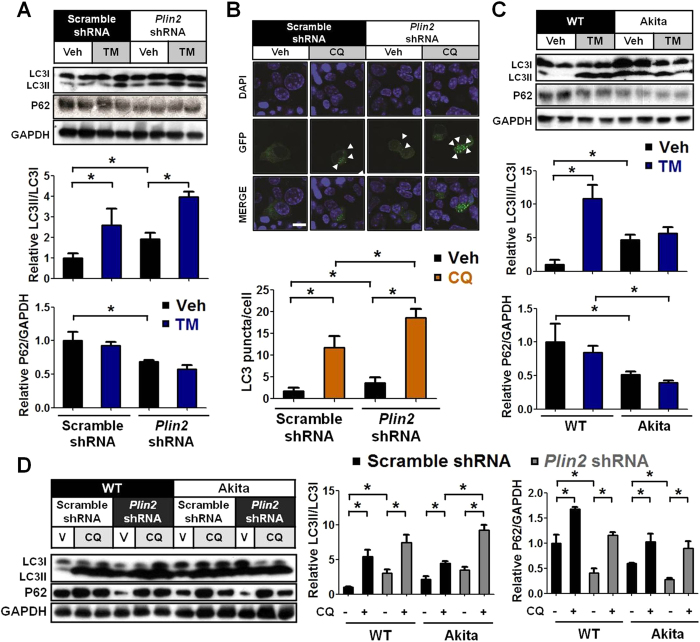
PLIN2 modulates autophagic flux in pancreatic β cells. (**A**) Immunoblotting and quantification. Control and *Plin2*-knockdown MIN6 cells were treated with vehicle or 5 μg/ml tunicamycin (TM) for 6 hours. GAPDH was used as loading control. (**B**) Control and *Plin2*-knockdown MIN6 cells were transfected with LC3-GFP construct and subquently treated with vehicle or 0.1 mM chloroquine (CQ) for 6 hours. Nuclei were stained with DAPI (blue). The number of LC3-GFP punctae (green dots) was quantified. Total of 50 GFP-positive cells were calculated each group. (**C**) Immunoblotting and quantification. Wild-type control and Akita β cells were treated with vehicle or 5 μg/ml TM for 6 hours. GAPDH was used as loading control. (**D**) Immunoblotting and quantification. Cells were treated with vehicle or 0.1 mM CQ for 6 hours. GAPDH was used as loading control. All immunoblotting quantification was normalized to scramble-shRNA vehicle. *p < 0.05. Scale bar = 5 μm.

Activated autophagy has been found to ameliorate ER stress^27^,^28^. We found that knockdown of *Plin2* in MIN6 cells raised the level of LC3II/LC3I and decreased the level of sequestosome 1 (SQSTM1/P62) (Fig. 7A), indicating that enhanced autophagy may underlie the improved ER stress in *Plin2*-deficient β cells. We transfected MIN6 cells with LC3-GFP and found an increased number of LC3 punctae in *Plin2*-knockdown compared to control cells. Addition of chloroquine (CQ), which inhibits the fusion of autophagosomes with lysosomes, led to an increased number of punctae, which remained significantly higher in *Plin2*-knockdown compared with control cells (Fig. 7B). These data support the interpretation that downregulation of PLIN2 stimulates autophagic flux in pancreatic β cells.

Under basal conditions, Akita cells displayed higher LC3II/LC3I ratio and lower P62 compared to wild-type cells (Fig. 7C). Compared with wild-type β cells, both chemical (TM) and pathophysiological (Akita) ER stress inducer led to a marked increase in LC3II/LC3I ratio and a decrease in P62, suggesting that increased ER stress leads to increased autophagic flux. We also noticed that TM treatment did not further boost the LC3II/LC3I ratio in Akita β cells, presumably because the increased autophagic flux was already at a saturated level under basal conditions (Fig. 7C).

We next examined the autophagic flux in the WT and Akita β cells with or without *Plin2* knockdown. *Plin2*-knockdown in wild-type or Akita β cells raised the level of LC3II/LC3I and decreased the level of P62 (Fig. 7D). Addition of CQ increased the LC3II/LC3I ratio and P62 levels in both scramble-shRNA and *Plin2*-knockdown wild-type and Akita cells (Fig. 7D). It is noteworthy that the LC3II/LC3I ratio was significantly higher in *Plin2*-knockdown than in scramble-shRNA Akita cells after CQ treatment (Fig. 7D).

## Discussion

In this study, we sought to determine the role of PLIN2 in modulating β cell function under ER stress conditions. We used mouse pancreatic islets, β cell lines and Akita mice to investigate the effects of PLIN2 under artificial and pathophysiological ER stress conditions and found that NEFA- and chemical-induced ER stress stimulated PLIN2 expression in β cells, and downregulation of PLIN2 ameliorated the deleterious effects of ER stress. Importantly, PLIN2 level was upregulated in β cells isolated from Akita mice, whose β cells exhibited severe ER stress because of failure of proinsulin processing. Genetic ablation of *Plin2* in Akita mice led to significant amelioration of hyperglycemia, decreased β cell apoptosis, and partially restored β cell mass and pancreatic insulin content. Finally, we found that the *Plin2*-deficient β cells display enhanced autophagic flux and accelerated ER stress resolution.

Many reports have shown that the PLIN2 expression level is correlated with intracellular lipid accumulation^10^,^11^,^12^,^13^. In fact, PLIN2 that is not associated with LD is reported to be rapidly degraded through the ubiquitin/proteasome pathway^29^. Interestingly, TM was found to stimulate intracellular lipid accumulation *in vitro* and *in vivo*^30^,^31^. Here, we also found that the intracellular TG levels in Akita and TM-treated cells are elevated compared with wild-type and vehicle controls, respectively. The increased TG levels may underlie the upregulated PLIN2 levels observed in Akita and TM-treated β cells. However, whether mechanisms independent of lipid accumulation, including mutant insulin and UPR-related activation, lead to increased PLIN2 remain to be explored.

Using MIN6 cells, antisense oligonucleotide, and human islets, Faleck *et al*. reported that PLIN2 is important in lipid metabolism and PA-stimulated insulin secretion in β cells^15^. Our study agrees with the findings that downregulation of PLIN2 decreases TG content in β cells. However, the effect of PA reported by Faleck *et al*. was acute (~1 h). It is in contrast to the effect of prolonged PA exposure which leads to ER stress^17^,^18^. Here, we show that downregulation of PLIN2 protects cells from prolonged NEFA exposure-induced ER stress. Further studies are required to investigate the complexity of PLIN2 function under different pathophysiological conditions.

Interestingly, using INS-1 β cells, Borg *et al*.^32^ reported that overexpression of PLIN1, a PLIN2-related protein, protects against β cell dysfunction after prolonged exposure to PA. PLIN1 is known to protect LDs from lipolysis by neutral lipases^8^; overexpressing PLIN1 may reduce lipolysis and hence prevent oxidative stress. Thus, there are different ways to overcome lipotoxicity. Furthermore, although PLIN1 and PLIN2 are LDPs that share limited structural similarities, their regulation, function and tissue distribution are very different^8^,^33^, and the two proteins may play very different roles in pancreatic β cells.

In our study, the ablation of *Plin2* in Akita mice partially reverses hyperglycemia and insulin deficiency. Interestingly, the partial preservation of β cell mass and improvement of hyperglycemia were also observed in different reports when *Cebpb, Chop*, or *Pik3r1* was deleted to prevent ER stress-induced β cell apoptosis^26^,^34^,^35^. Importantly, treatment of Akita mice with rapamycin, which induces autophagy, also led to a partial restoration of pancreatic insulin content compared with vehicle-treated Akita mice^36^. Our study and other reports suggest that induced autophagy and reduced β cell apoptosis partially ameliorate, but do not completely reverse, the severe phenotype in Akita mice.

Basal autophagy is important for the maintenance of normal pancreatic islet architecture and function^37^,^38^,^39^. Many studies have found that properly activated autophagy helps maintain ER homeostasis and protects against excessive ER stress^27^,^28^,^38^,^40^. Especially relevant to this study is the fact that chemical-mediated stimulation of autophagy was found to improve ER stress-induced diabetes in Akita mice^36^. Importantly, we showed that pancreatic β cells isolated from Akita mice display increased basal autophagy compared to wild-type control cells, which may function as a protective response to the persistent ER stress in Akita β cells. Unlike wild-type cells, which respond to TM by increased autophagy, the pancreatic β cells in Akita mice in the basal state appeared to already exhibit maximal autophagic flux, which failed to be further augmented by TM treatment (Fig. 7C). Interestingly, however, the autophagy capacity could be further enhanced when we knocked down *Plin2* in both wild-type and Akita β cells (Fig. 7D).

The persistent basal ER stress in Akita β cells causes cellular lipids as well as PLIN2 to accumulate, as observed in other types of cells^41^. The observation that Akita β cells exhibit increased autophagy in the backdrop of elevated PLIN2 may seem paradoxical. It is noteworthy, however, that there are multiple pathways by which a cell can activate autophagy, e.g., the UPR signaling pathways IRE1-JNK and PERK-eIF2α have both been shown to mediate LC3 conversion independently and play a pivotal role in ER stress-induced autophagy^42^,^43^. In this study, we found that induced PLIN2 downregulation stimulates autophagic flux, whereby protecting the Akita;*Plin2*^−/−^ β cells from the deleterious effects of inducer-activated ER stress in Akita mice. Herein, we have uncovered a previously unappreciated role of PLIN2 in modulating autophagy, ER stress resolution, and β cell survival.

## Additional Information

**How to cite this article**: Chen, E. *et al*. PLIN2 is a Key Regulator of the Unfolded Protein Response and Endoplasmic Reticulum Stress Resolution in Pancreatic β Cells. *Sci. Rep.* **7**, 40855; doi: 10.1038/srep40855 (2017).

**Publisher's note:** Springer Nature remains neutral with regard to jurisdictional claims in published maps and institutional affiliations.

## Supplementary Material

Supplementary Figure S1

## References

[b1] Schuit F. C., Kiekens R. & Pipeleers D. G. Measuring the balance between insulin synthesis and insulin release. Biochem. Biophys. Res. Commun. 178, 1182–1187 (1991). 1872837 10.1016/0006-291x(91)91017-7

[b2] Harding H. P. & Ron D. Endoplasmic reticulum stress and the development of diabetes: a review. Diabetes 51, Suppl 3, S455–61 (2002). 12475790 10.2337/diabetes.51.2007.s455

[b3] Back S. H. & Kaufman R. J. Endoplasmic reticulum stress and type 2 diabetes. Annu. Rev. Biochem. 81, 767–793 (2012). 22443930 10.1146/annurev-biochem-072909-095555 PMC3684428

[b4] Laybutt D. R. . Endoplasmic reticulum stress contributes to beta cell apoptosis in type 2 diabetes. Diabetologia 50, 752–763 (2007). 17268797 10.1007/s00125-006-0590-z

[b5] Huang C. J. . High expression rates of human islet amyloid polypeptide induce endoplasmic reticulum stress mediated beta-cell apoptosis, a characteristic of humans with type 2 but not type 1 diabetes. Diabetes 56, 2016–2027 (2007). 17475933 10.2337/db07-0197

[b6] Marchetti P. . The endoplasmic reticulum in pancreatic beta cells of type 2 diabetes patients. Diabetologia 50, 2486–2494 (2007). 17906960 10.1007/s00125-007-0816-8

[b7] Boden G. & Shulman G. I. Free fatty acids in obesity and type 2 diabetes: defining their role in the development of insulin resistance and beta-cell dysfunction. Eur. J. Clin. Invest. 32 Suppl 3, 14–23 (2002). 12028371 10.1046/j.1365-2362.32.s3.3.x

[b8] Bickel P. E., Tansey J. T. & Welte M. A. PAT proteins, an ancient family of lipid droplet proteins that regulate cellular lipid stores. Biochim. Biophys. Acta 1791, 419–440 (2009). 19375517 10.1016/j.bbalip.2009.04.002 PMC2782626

[b9] Larsson S., Resjo S., Gomez M. F., James P. & Holm C. Characterization of the lipid droplet proteome of a clonal insulin-producing beta-cell line (INS-1 832/13). J. Proteome Res. 11, 1264–1273 (2012). 22268682 10.1021/pr200957p

[b10] Gao J. & Serrero G. Adipose differentiation related protein (ADRP) expressed in transfected COS-7 cells selectively stimulates long chain fatty acid uptake. J. Biol. Chem. 274, 16825–16830 (1999). 10358026 10.1074/jbc.274.24.16825

[b11] Imamura M. . ADRP stimulates lipid accumulation and lipid droplet formation in murine fibroblasts. Am. J. Physiol. Endocrinol. Metab. 283, E775–83 (2002). 12217895 10.1152/ajpendo.00040.2002

[b12] Dalen K. T., Ulven S. M., Arntsen B. M., Solaas K. & Nebb H. I. PPARalpha activators and fasting induce the expression of adipose differentiation-related protein in liver. J. Lipid Res. 47, 931–943 (2006). 16489205 10.1194/jlr.M500459-JLR200

[b13] Chang B. H. . Protection against fatty liver but normal adipogenesis in mice lacking adipose differentiation-related protein. Mol. Cell. Biol. 26, 1063–1076 (2006). 16428458 10.1128/MCB.26.3.1063-1076.2006 PMC1347045

[b14] Chang B. H., Li L., Saha P. & Chan L. Absence of adipose differentiation related protein upregulates hepatic VLDL secretion, relieves hepatosteatosis, and improves whole body insulin resistance in leptin-deficient mice. J. Lipid Res. 51, 2132–2142 (2010). 20424269 10.1194/jlr.M004515 PMC2903828

[b15] Faleck D. M. . Adipose differentiation-related protein regulates lipids and insulin in pancreatic islets. Am. J. Physiol. Endocrinol. Metab. 299, E249–57 (2010). 20484013 10.1152/ajpendo.00646.2009 PMC2928510

[b16] Akerfeldt M. C. . Cytokine-induced beta-cell death is independent of endoplasmic reticulum stress signaling. Diabetes 57, 3034–3044 (2008). 18591394 10.2337/db07-1802 PMC2570400

[b17] Karaskov E. . Chronic palmitate but not oleate exposure induces endoplasmic reticulum stress, which may contribute to INS-1 pancreatic beta-cell apoptosis. Endocrinology 147, 3398–3407 (2006). 16601139 10.1210/en.2005-1494

[b18] Kharroubi I. . Free fatty acids and cytokines induce pancreatic beta-cell apoptosis by different mechanisms: role of nuclear factor-kappaB and endoplasmic reticulum stress. Endocrinology 145, 5087–5096 (2004). 15297438 10.1210/en.2004-0478

[b19] Stoy J. . Insulin gene mutations as a cause of permanent neonatal diabetes. Proc. Natl. Acad. Sci. USA 104, 15040–15044 (2007). 17855560 10.1073/pnas.0707291104 PMC1986609

[b20] Ahamed A. . Permanent neonatal diabetes mellitus due to a C96Y heterozygous mutation in the insulin gene. A case report. JOP 9, 715–718 (2008). 18981553

[b21] Yang Y. & Chan L. Monogenic Diabetes: What It Teaches Us on the Common Forms of Type 1 and Type 2 Diabetes. Endocr. Rev. 37, 190–222 (2016). 27035557 10.1210/er.2015-1116 PMC4890265

[b22] Yoshioka M., Kayo T., Ikeda T. & Koizumi A. A novel locus, Mody4, distal to D7Mit189 on chromosome 7 determines early-onset NIDDM in nonobese C57BL/6 (Akita) mutant mice. Diabetes 46, 887–894 (1997). 9133560 10.2337/diab.46.5.887

[b23] Wang J. . A mutation in the insulin 2 gene induces diabetes with severe pancreatic beta-cell dysfunction in the Mody mouse. J. Clin. Invest. 103, 27–37 (1999). 9884331 10.1172/JCI4431 PMC407861

[b24] Nozaki J. . The endoplasmic reticulum stress response is stimulated through the continuous activation of transcription factors ATF6 and XBP1 in Ins2+/Akita pancreatic beta cells. Genes Cells 9, 261–270 (2004). 15005713 10.1111/j.1356-9597.2004.00721.x

[b25] Nakagawa T. . Caspase-12 mediates endoplasmic-reticulum-specific apoptosis and cytotoxicity by amyloid-beta. Nature 403, 98–103 (2000). 10638761 10.1038/47513

[b26] Matsuda T. . Ablation of C/EBPbeta alleviates ER stress and pancreatic beta cell failure through the GRP78 chaperone in mice. J. Clin. Invest. 120, 115–126 (2010). 19955657 10.1172/JCI39721 PMC2798684

[b27] Bernales S., McDonald K. L. & Walter P. Autophagy counterbalances endoplasmic reticulum expansion during the unfolded protein response. PLoS Biol. 4, e423 (2006). 17132049 10.1371/journal.pbio.0040423 PMC1661684

[b28] Ogata M. . Autophagy is activated for cell survival after endoplasmic reticulum stress. Mol. Cell. Biol. 26, 9220–9231 (2006). 17030611 10.1128/MCB.01453-06 PMC1698520

[b29] Xu G. . Post-translational regulation of adipose differentiation-related protein by the ubiquitin/proteasome pathway. J. Biol. Chem. 280, 42841–42847 (2005). 16115879 10.1074/jbc.M506569200

[b30] Rutkowski D. T. . UPR pathways combine to prevent hepatic steatosis caused by ER stress-mediated suppression of transcriptional master regulators. Dev. Cell. 15, 829–840 (2008). 19081072 10.1016/j.devcel.2008.10.015 PMC2923556

[b31] Kim A. J., Shi Y., Austin R. C. & Werstuck G. H. Valproate protects cells from ER stress-induced lipid accumulation and apoptosis by inhibiting glycogen synthase kinase-3. J. Cell. Sci. 118, 89–99 (2005). 15585578 10.1242/jcs.01562

[b32] Borg J. . Perilipin is present in islets of Langerhans and protects against lipotoxicity when overexpressed in the beta-cell line INS-1. Endocrinology 150, 3049–3057 (2009). 19299455 10.1210/en.2008-0913 PMC2703509

[b33] Londos C., Sztalryd C., Tansey J. T. & Kimmel A. R. Role of PAT proteins in lipid metabolism. Biochimie 87, 45–49 (2005). 15733736 10.1016/j.biochi.2004.12.010

[b34] Oyadomari S. . Targeted disruption of the Chop gene delays endoplasmic reticulum stress-mediated diabetes. J. Clin. Invest. 109, 525–532 (2002). 11854325 10.1172/JCI14550 PMC150879

[b35] Winnay J. N., Dirice E., Liew C. W., Kulkarni R. N. & Kahn C. R. P85alpha Deficiency Protects Beta-Cells from Endoplasmic Reticulum Stress-Induced Apoptosis. Proc. Natl. Acad. Sci. USA 111, 1192–1197 (2014). 24395790 10.1073/pnas.1322564111 PMC3903202

[b36] Bachar-Wikstrom E. . Stimulation of autophagy improves endoplasmic reticulum stress-induced diabetes. Diabetes 62, 1227–1237 (2013). 23274896 10.2337/db12-1474 PMC3609555

[b37] Jung H. S. . Loss of autophagy diminishes pancreatic beta cell mass and function with resultant hyperglycemia. Cell. Metab. 8, 318–324 (2008). 18840362 10.1016/j.cmet.2008.08.013

[b38] Ebato C. . Autophagy is important in islet homeostasis and compensatory increase of beta cell mass in response to high-fat diet. Cell. Metab. 8, 325–332 (2008). 18840363 10.1016/j.cmet.2008.08.009

[b39] Riahi Y. . Autophagy is a major regulator of beta cell insulin homeostasis. Diabetologia 59, 1480–1491 (2016). 26831301 10.1007/s00125-016-3868-9 PMC5912938

[b40] Choi S. E. . Protective role of autophagy in palmitate-induced INS-1 beta-cell death. Endocrinology 150, 126–134 (2009). 18772242 10.1210/en.2008-0483

[b41] Basseri S. & Austin R. C. Endoplasmic reticulum stress and lipid metabolism: mechanisms and therapeutic potential. Biochem. Res. Int. 2012, 841362 (2012). 22195283 10.1155/2012/841362 PMC3238353

[b42] Kouroku Y. . ER stress (PERK/eIF2alpha phosphorylation) mediates the polyglutamine-induced LC3 conversion, an essential step for autophagy formation. Cell Death Differ. 14, 230–239 (2007). 16794605 10.1038/sj.cdd.4401984

[b43] Ding W. X. . Linking of autophagy to ubiquitin-proteasome system is important for the regulation of endoplasmic reticulum stress and cell viability. Am. J. Pathol. 171, 513–524 (2007). 17620365 10.2353/ajpath.2007.070188 PMC1934546

