# Supplementary material for: PLIN2 is a Key Regulator of the Unfolded Protein Response and Endoplasmic Reticulum Stress Resolution in Pancreatic β Cells
Source: Sci Rep. 2017 Jan 19;7:40855. doi: 10.1038/srep40855 (PMC5244387; doi:10.1038/srep40855)
Supplement: Supplementary Figure S1 [file srep40855-s1.doc]

**PLIN2 is a Key Regulator of the Unfolded Protein Response and**

**Endoplasmic Reticulum Stress Resolution in Pancreatic β Cells**

Elaine Chen^1^, Tsung Huang Tsai^2^, Lan Li^2^, Pradip Saha^1^,

Lawrence Chan ^1,2^, and Benny Hung-Junn Chang ^1,2^

^1^ Department of Molecular & Cellular Biology

^2^ Department of Medicine, Division of Diabetes, Endocrinology & Metabolism, Diabetes Research Center

Baylor College of Medicine, 1 Baylor Plaza, Houston, TX 77030

Corresponding authors:

**Lawrence Chan** ([lchan@bcm.edu](mailto:lchan@bcm.edu)) and **Benny Hung-Junn Chang** ([bchang@bcm.edu](mailto:bchang@bcm.edu))

address: 1 Baylor Plaza, R614, Houston, TX 77030

tel: 713-798-6686

fax: 713-798-8764


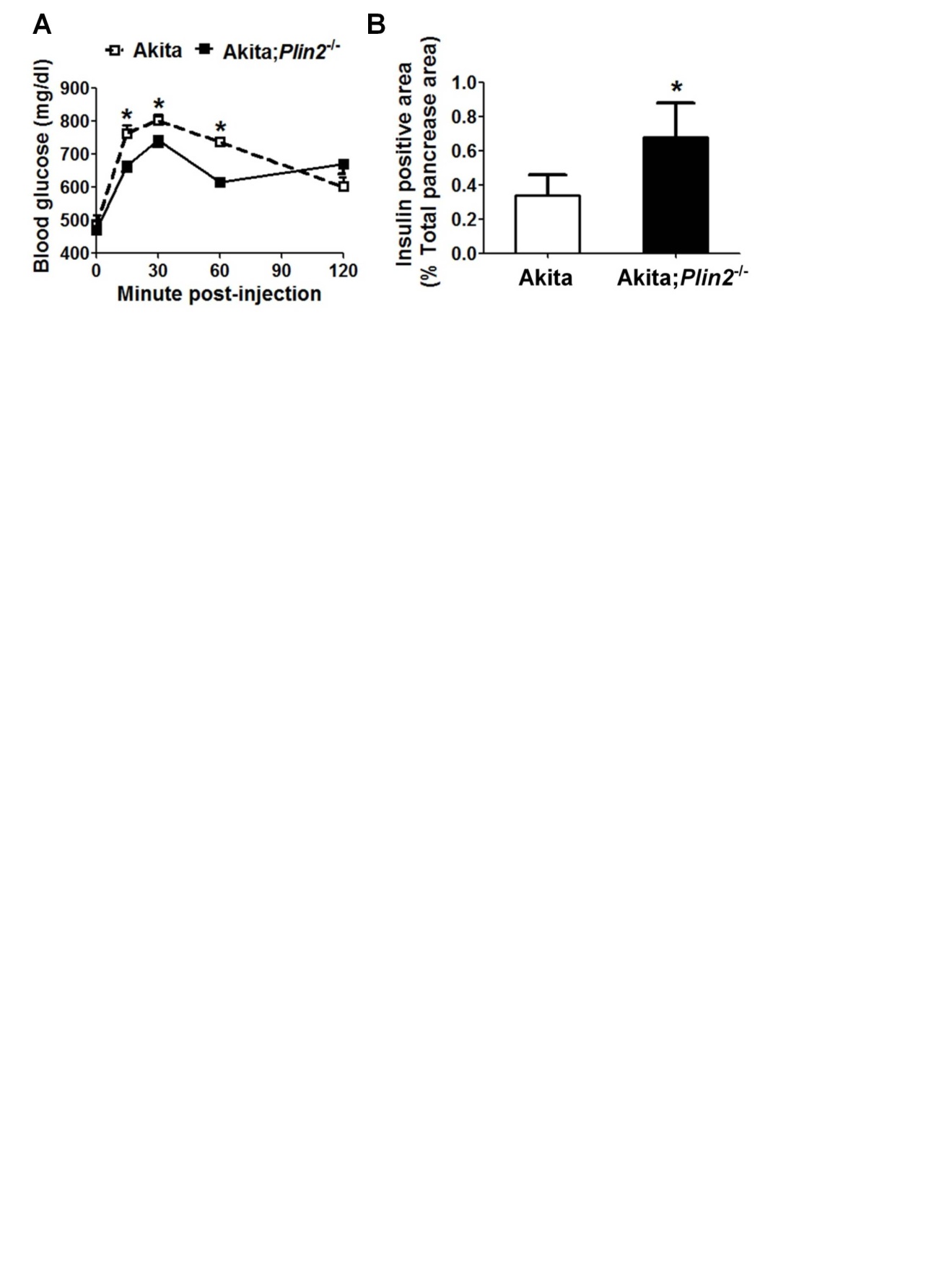


**Figure S1. Ablation of PLIN2 improves glucose tolerance test response and rescues β cell mass in Akita mice.**

(**A**) Blood glucose levels at designated time points during intraperitoneal glucose tolerance test of 12-week-old male mice. Mice were fasted for 4 hours in the morning prior to injection with 1.5 g glucose/kg body weight. (**B**) Quantification of insulin positive area relative to the total pancreas area. Insulin-positive and total pancreas areas were computed from 6 sections per mouse (n = 5). *, *p*<0.05.
